# A case series of dupilumab for vitiligo and alopecia areata in the setting of atopic dermatitis

**DOI:** 10.1016/j.jdcr.2025.05.048

**Published:** 2025-07-15

**Authors:** Dev Patel, Ben Hu, Omar Alani, Nanette Silverberg

**Affiliations:** Department of Dermatology, Icahn School of Medicine at Mount Sinai, New York, New York

**Keywords:** alopecia areata, asthma, atopic dermatitis, dupilumab, IL-4/IL-13 signaling, IL-4R alpha subunit, Koebner phenomenon, melanocytes, monoclonal antibody, refractory pruritus, repigmentation, Th2 cytokines, vitiligo

## Introduction

Dupilumab is a human monoclonal antibody of the IgG4 subclass and binds to the interleukin (IL)-4R alpha subunit, causing downstream inhibition of IL-4 and IL-13 signaling, downregulating the T-helper-2 cytokine response.^1^ Dupilumab is FDA-approved to treat atopic dermatitis (AD) (moderate-to-severe), asthma (moderate-to-severe), chronic rhinosinusitis with nasal polyposis, and eosinophilic esophagitis. Vitiligo is a common autoimmune depigmenting skin disorder observed in 1.38% of US adults, and up to 2.16% of adolescents in the US, with a range of 0.4% to 2% in most populations.^2^, ^3^, ^4^ Vitiligo is an autoimmune destruction of melanocytes, with the participation of keratinocytes in the destructive process. The Koebner phenomenon in vitiligo occurs when trauma induces new lesions, with events including scratching as would be noted in AD.^5^ AD has also been linked to vitiligo, especially in children under the age of 12 years.^6^ There are many available therapies for vitiligo, including topical agents, systemic agents, phototherapy, and surgical repigmentation.^7^^,^^8^ Alopecia areata (AA) has also been identified as a comorbidity of AD. We sought to characterize the potential benefits of dupilumab on AA and vitiligo in individuals who overlap with AD.^9^ An IRB-exempt review was conducted of patient charts for individuals who received dupilumab and who had AA or vitiligo associated with AD.

## Case series

**Fig 1:**
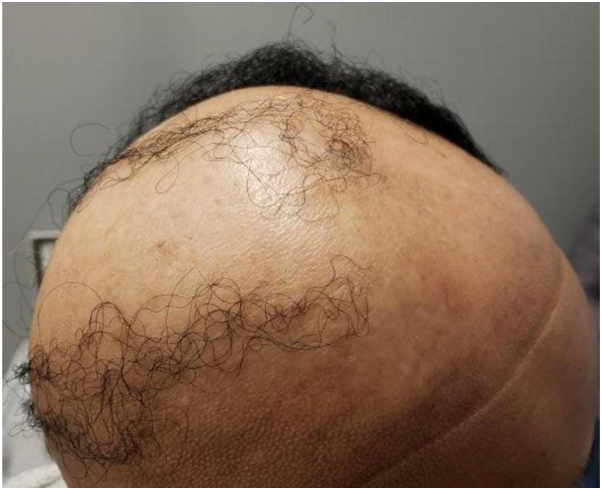
A 24 year-old African American female with atopic dermatitis (AD) and alopecia universalis for 18 years before therapy with dupixent 600 mg loading dosage and 300 mg every 2 weeks subcutaneously thereafter.

**Fig 2:**
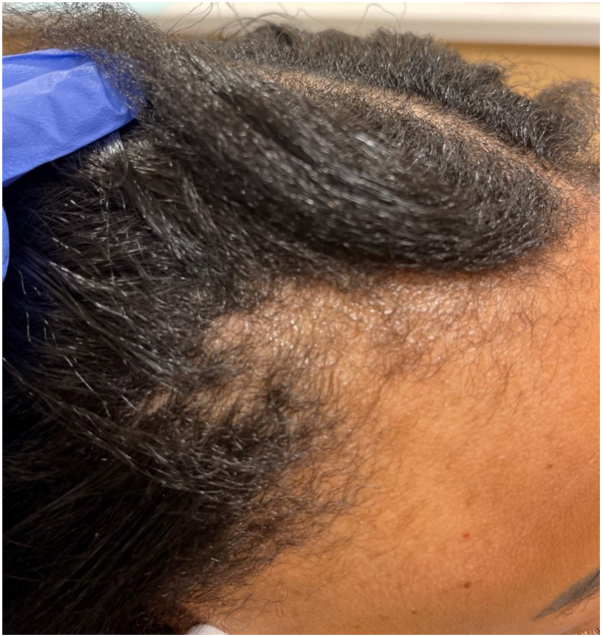
The same patient featured in Fig 1 after 1 years of dupilumab usage.

Six patients with AD and alopecia universalis (AU), and 1 patient with AD, vitiligo, and AA were identified for review. The patients treated for AU are summarized in Table I and include a 5-year-old Asian female, a 12-year-old white female, a 14-year-old African American male, a 15-year-old Hispanic female, a 24-year-old African-American female, and a 78-year-old Hispanic female. The five-year-old regrew hair for 4 months but had a rapid loss when forced to discontinue due to insurance coverage alterations. The 12-year-old female who had AU for 8 years had no regrowth. The 14-year-old regrew his hair and experienced some conjunctivitis briefly after 12 months, at which time his eyelashes had just regrown. All of the patients with comorbid AD achieved EASI-75 at 6 months. The 24-year-old (Figs 1 and 2, before and after) and 78-year-old had rapid regrowth of hair.

**Table I:** Summary of cases of alopecia areata and vitiligo treated with dupilumab

Diagnosis	Age of the patient	Length of disease	Race/ethnicity	Sex	Clinical course	Prior drugs failed	Comorbidities
Alopecia universalis	5-y-old	6 mo	Asian	Female	Regrew (80%) with 4 mo, but lost hair shortly after stopping	Topical clobetasol and betamethasone	Atopic dermatitis
Alopecia universalis	12-y-old	8 y	White	Female	No regrowth after 6 mo	Topical clobetasol, intralesional corticosteroids, anthralin, topical squaric acid dibutyl ester	Atopic dermatitis
Alopecia universalis	14-y-old	6 y	African American	Male	Regrew completely over 14 mo; developed conjunctivitis once his eyelashes grew	Topical clobetasol, topical squaric acid, 308 nm laser	Atopic dermatitis
Alopecia universalis	15-y-old	2 y	Hispanic	Female	Regrew completely, shed hair after 8 mo, then regrew with the addition of oral ritlecitinib	Intralesional corticosteroids	Atopic dermatitis
Alopecia universalis	24-y-old	16 y	African American	Female	Regrew hair over central 70% of the scalp in 1 y, required an additional year to regrow the ophiasis region	Intralesional corticosteroids, clobetasol, minoxidil, anthraling, squaric acid dibutyl ester	Atopic dermatitis
Alopecia universalis	78-y-old	30 y	Hispanic	Female	Regrew hair starting 3 mo into start and then continued to regrow over 16 mo	Intralesional corticosteroids	Asthma
Alopecia totalis and vitiligo	61-y-old	4 y	African American	Female	Rapid repigmentation and hair regrowth	Topical corticosteroids	Hypertension

The 15-year-old regrew completely and then shed hair, requiring the addition of oral ritlecitinib 50 mg per day, which promoted subtotal regrowth of hair. The patient tried coming off dupixent after her hair regrew but restarted due to flaring of her AD.

**Fig 3:**
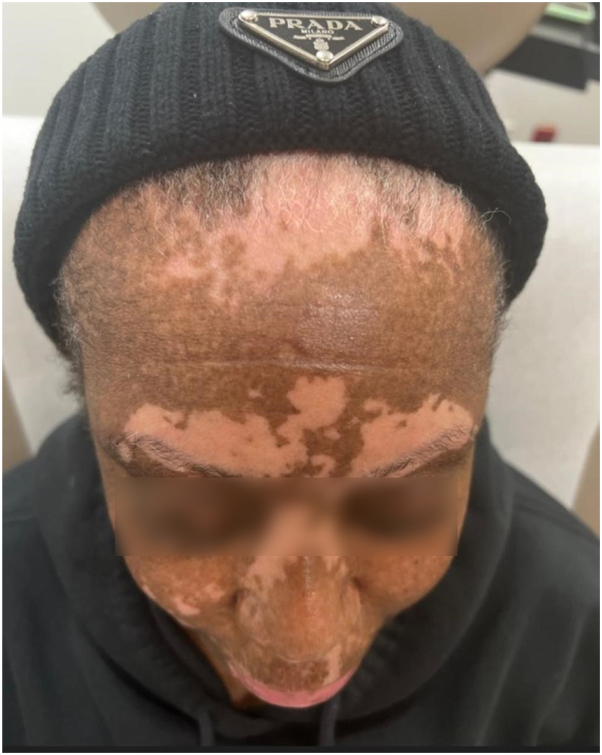
A 61-year-old African American female with atopic dermatitis (AD), alopecia areata (AA), and vitiligo before starting therapy with dupixent 600 mg loading dosage and 300 mg every 2 weeks subcutaneously thereafter.

**Fig 4:**
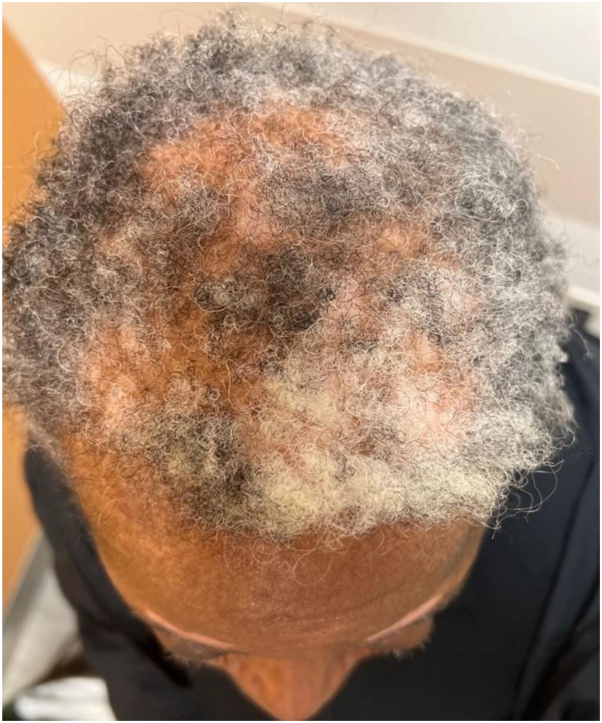
The same patient featured in Fig 3 after 1 year of combination dupilumab and topical 1.5% ruxolitinib for the facial lesions.

The vitiligo/AA patient is a 61-year-old woman with 50% BSA confetti lesion vitiligo affecting the chest, back, abdomen, arms, and legs. She had rapid disease stabilization (2 months) and at 1 year had 90% facial and 70% extremity repigmentation with dupilumab and the addition of topical 1.5% ruxolitinib for facial disease. The same patient had 40% scalp hair loss which fully resolved upon repigmentation of the scalp. The response was noted rapidly but plateaued at 12 months (Figs 3 and 4, before and after).

## Discussion

The overlap of AD with AA and vitiligo points to a shared pathogenesis. One of the likely reasons for this is the Koebner phenomenon. We hypothesize that the Koebner phenomenon is triggered through an IL-4/IL-13 mechanism, i.e. for specific individuals IL-4 and IL-13 hyper-reactivity can be a Koebner-based trigger. Additionally, AA overlaps with AD and is poorly characterized. However, the linkage has been recognized in recent AAD guidelines addressing AD comorbidities.^9^ Checkpoint inhibition is a potential theoretical pathway as well, which has been poorly characterized but has been implicated in AD and vitiligo.^10^

We hypothesize that IL-4/IL-13 hyper-reactivity in the skin can act similarly in AU. There is in AA is an overlap of TH1-CXCL9/10 expression and interferon-gamma overexpression in addition to Th2- including IL-13 overexpression.^11^ Therefore, the blockade of IL-4/IL-13 may be effective through multiple mechanisms of activity. There is already notable Phase 2a data supporting usage of dupilumab in AA.^12^ Our experience demonstrates sustained hair growth in half of the patients treated. AA/vitiligo overlap appears to respond well to dupilumab. This is supported by a recent case report demonstrating the benefit of dupilumab in programmed cell death inhibitor-1-induced vitiligo with associated refractory pruritus.^13^ Liu et al have observed in vitro that rising IL-4 levels were linked to increased vitiligo risk.^14^ On the other hand, some reports of vitiligo after initiating dupilumab therapy do exist, with some new-onset and worsening described. These cases are limited but bear consideration. In our patient, disease stabilization was noted in a generalized confetti-vitiligo patient, but a topical Janus kinase inhibitor was used adjunctively.^15^ Given the need for long-term maintenance, the safety of dupilumab is ideal. Therefore, further exploration of dupilumab therapy for vitiligo/AA/AD, vitiligo/AD/Koebner phenomenon positive, and AU/AD is needed. This is particularly important to address in patients under the age of 12 years who currently have no approved systemic medications for vitiligo and AU. When AU, vitiligo, and combinations of the two are comorbid with AD, there is an expectation of circulating IL-4/13 elevations and localized IL-4/13 elevation that support a potential role for dupilumab therapy in these conditions. Given that AD is associated with vitiligo of childhood and severe AA is linked to AD, systemic therapy would be beneficial in these individuals.

## Conflicts of interest

None disclosed.
